# Personalizing Radiation Treatment Delivery in the Management of Breast Cancer

**DOI:** 10.1155/2018/6729802

**Published:** 2018-06-10

**Authors:** Kamran A. Ahmed, G. Daniel Grass, Amber G. Orman, Casey Liveringhouse, Michael E. Montejo, Hatem H. Soliman, Heather S. Han, Brian J. Czerniecki, Javier F. Torres-Roca, Roberto Diaz

**Affiliations:** ^1^Department of Radiation Oncology, H. Lee Moffitt Cancer Center and Research Institute, Tampa, FL 33612, USA; ^2^University of South Florida College of Medicine, Tampa, FL 33612, USA; ^3^Department of Breast Oncology, H. Lee Moffitt Cancer Center and Research Institute, Tampa, FL 33612, USA

## Abstract

Long-term data establishes the efficacy of radiotherapy in the adjuvant management of breast cancer. New dose and fractionation schemas have evolved and are available, each with unique risks and rewards. Current efforts are ongoing to tailor radiotherapy to the unique biology of breast cancer. In this review, we discuss our efforts to personalize radiotherapy dosing using genomic data and the implications for future clinical trials. We also explore immune mechanisms that may contribute to a tumor's unique radiation sensitivity or resistance.

## 1. Introduction

Radiotherapy is integral in the management of breast cancer. The 25-year results of NSABP B04 published in 2002 indicate radiation leads to less extensive surgeries, while maintaining relapse-free and overall survival [1]. Meta-analyses show that locoregional control as well as breast cancer mortality benefit from adjuvant radiation therapy following breast conservation surgery or following mastectomy with node-positive disease [2, 3].

Technical advances in radiation therapy treatment planning since these early studies including target motion management, image guidance, and conformal planning now result in improved ability to decrease dose to surrounding organs including the heart and lungs while accurately treating diseased tissue [4, 5]. Also a number of radiation fractionation strategies are now validated for both early and advanced stage breast cancer. Hypofractionation studies have revealed equivalent treatment outcomes with respect to in-breast local control, breast cosmesis, and toxicity while being more convenient and cost-effective with shortened treatment duration[6–8]. Partial breast irradiation via external beam, brachytherapy, or intraoperative techniques has been shown to limit the volume of irradiated tissue in select groups of women while preserving efficacy although data on long-term outcomes is limited [9–12].

Personalized medicine has been discussed in the medical oncology community; however, radiation oncologists have typically delivered uniform doses of radiation without consideration to biologic differences across tumors. As we enter a new era of genomic testing, personalized radiation therapy is becoming more feasible. Breast cancer is well suited to be a primary malignancy in which radiation oncologists' pilot efforts for personalization. The role of adjuvant radiation therapy is well established in breast cancer and gene expression data for breast malignances exists in large publicly available datasets. For this reason, our group has focused attention on personalizing radiation therapy in this malignancy.

In this review, we will discuss efforts to define the radiation sensitivity and resistance of genomically characterized breast tumor types as determined by our novel gene expression based radiosensitivity index (RSI). We also discuss personalized radiotherapy dosing after interpretation of RSI as well as development of an actionable radiation metric, the genomically adjusted radiation dose (GARD). Initial validation studies in breast tumors using this model will be reviewed, and we will also discuss rational combinations of radiation therapy with immunotherapy utilizing RSI. Finally, we will close with thoughts on prospective trials to test these hypotheses.

## 2. Biologic Adaptation in Systemic Management of Breast Cancer

Genomic tests have become useful tools in predicting clinical outcomes and for guiding treatment decisions in breast cancer, particularly for systemic therapy. For example, the Mammaprint 70-gene signature test has been shown to be a powerful predictor of distant metastasis in node-negative breast cancer, and there is evidence it is a more accurate predictor of overall survival and metastasis-free survival than standard clinicopathological risk assessment methods [13, 14]. Traditionally, women considered to be at high clinical risk for distant metastasis were treated with adjuvant or neoadjuvant chemotherapy. However, approximately 46% of these women may not require chemotherapy if they are placed in the low genomic risk category by the Mammaprint signature as these women were found to gain no benefit in five-year metastasis-free survival with adjuvant chemotherapy [15].

The Oncotype Dx recurrence score was shown to be an accurate predictor for the risk of distant recurrence and overall survival at 10 years independent of age and tumor size in patients with lymph node-negative, ER+ tumors treated with tamoxifen [16]. In lymph node-positive patients treated with tamoxifen, adjuvant chemotherapy provided little benefit in terms of 10-year distant recurrence in tumors with Oncotype Dx recurrence scores of less than 18, while patients with recurrence scores greater than 31 benefited from chemotherapy [17, 18]. Furthermore, prospective studies showed patients with node-negative, ER+, HER2-negative tumors and recurrence scores <11 had less than a 1% risk of distant recurrence at five years and that such patients may be safely spared chemotherapy even if they are at high risk of distant failure by traditional clinicopathologic risk estimation [19, 20].

With growing acceptance of data supporting the use of tumor genomics in clinical practice, the newly released AJCC 8^th^ edition staging guidelines will incorporate tumor genomic assays as staging modifiers. For example, in hormone receptor positive, HER2-negative, lymph node-negative tumors, a low risk score on the Mammaprint signature or a recurrence score less than 11 on the Oncotype Dx panel places a tumor into the same prognostic category as T1a-T1b N0 M0, regardless of tumor size [21]. While tumor genomic panels have seen an increased use in guiding systemic therapy decisions, similar tests have not gained widespread adoption in radiotherapy management of breast cancer.

## 3. Biologic Adaptation in Breast Radiotherapy

Efforts are currently underway in the breast radiation oncology community to tailor adjuvant treatment to a patient's biologic subtype. Although the role of radiation treatment is known in the advanced node-positive setting, in localized tumors with other favorable prognostic characteristics there is an understanding that as radiation oncologists we may be overtreating patients who may otherwise be eligible for systemic treatment alone. Trials omitting radiation in select favorable patients have shown higher rates of recurrences indicating improved techniques to select appropriate patients for treatment deescalation are needed. The 10-year results from the CALGB 9343 study have revealed in women ≥ 70 years with T1, node-negative tumors, that are ER+ and receiving hormonal therapy, radiation therapy can be eliminated after lumpectomy with fairly low recurrence rates [22, 23]. The CALGB study demonstrated a freedom from locoregional recurrence rate of 90% in the lumpectomy with tamoxifen alone treated arm and 98% in the arm treated with radiotherapy. The difference was significant; however, OS did not differ between groups. In addition, the PRIME II trial has demonstrated the feasibility of eliminating radiation in a cohort of women ≥ 65 years, pN0, up to 3 cm tumors and negative margins who received adjuvant endocrine therapy [24]. At 5 years, recurrence rates in the arm without radiation were significantly higher at 4.1% compared to 1.3%; however, the study requires continued long-term follow-up.

Given these trials, which have demonstrated the feasibility of excluding radiation therapy in the management of early breast tumors without decreasing overall survival, a number of studies are ongoing to assess which patients may be candidates for radiation therapy exclusion. The LUMINA study from the Ontario Clinical Oncology Group (Clinicaltrials.gov identifier NCT01791829) is a single arm study of women ≥ 55 years with T1 luminal A tumors receiving endocrine therapy. In addition, the University of Michigan has initiated the multi-institutional IDEA trial (NCT02400190) in which women between 50 and 69 years with ER+, PR+, and Her 2 negative early stage tumors with an Oncotype Dx score ≤ 18 will receive hormonal therapy alone. Finally, the Dana Farber Cancer Institute has initiated the precision trial for women between the ages of 50 and 75 years with luminal A tumors measuring ≤ 2 cm that receive hormonal therapy alone. Together, these efforts are targeting more specific populations of women utilizing biologic subtype to better characterize which women can be spared radiation therapy. Although there are ongoing efforts to personalize radiation therapy delivery based on a patient's unique clinical factors as well as receptor status, genomic data would be expected to vary amongst these tumors. Efforts from our group as well as others have suggested methods may exist to tailor radiation therapy based on a tumor's unique genomic profile.

Various groups have suggested utilization of gene signatures and biomarkers to predict the benefit of radiation therapy in both early and advanced stage breast cancer. The Danish Group published a gene signature, which predicted the benefit of postmastectomy RT in patients with high-risk breast cancer in the context of the Danish 82b and 82c trials [25]. A seven-gene signature was identified from 191 patients and then validated in 112 patients ultimately identifying a group of patients with sufficiently low risk of locoregional recurrence in whom there was no benefit from postmastectomy radiation therapy [26, 27]. Similarly, studies have revealed the Oncotype Dx score to be predictive of locoregional recurrence suggesting potential utility in radiotherapy treatment decision-making. The University of Michigan has also suggested a radiation sensitivity signature (RSS) to identify patients that would benefit from adjuvant radiotherapy [28]. The RSS was developed using clonogenic survival assays across breast cancer cell lines. The RSS was refined to 51 genes and validated in two independent datasets outperforming all clinical and pathologic features.

**Figure 1:**
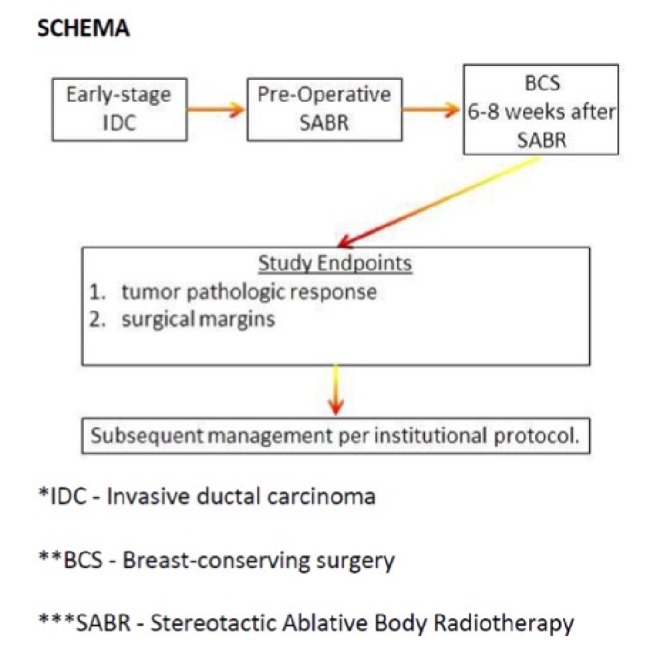
Trial schema for preoperative stereotactic radiation trial.

To better understand the effects of radiation therapy and the tumor microenvironment we have recently opened a phase 2 trial at our institution to assess a preoperative accelerated partial breast irradiation (APBI) regimen of 28.5 Gy in 3 fractions following 6 to 8 weeks later by surgical resection (Figure 1). Although several attempts at preoperative radiation have occurred in the phase I setting including 21 Gy in one fraction and 31.5 Gy in three fractions [29, 30], an important goal of our trial is to obtain pre- and postradiated tissue to assess the unique changes in the radiation sensitivity and to assess the immune landscape that may be important to the tumor's sensitivity to radiation.

## 4. RSI Development and Validation

Historically, radiation therapy dose delivery and fractionation schemes have been uniform with variation only in definition of target volume (i.e., +/- nodal volumes). A patient's unique tumor biology has not been taken into account. The development and validation of the radiosensitivity index (RSI) have taken place over the past decade as a means to help predict radiosensitivity of various tumor types and response to radiation treatment [31]. RSI was developed to predict differences in cellular radiosensitivity based on the surviving fraction of cells at 2 Gy (SF2) in cell lines. The process of developing the signature included two main steps. The first step included the identification of 10 genes using an algorithm correlating basal gene expression and other parameters (tissue of origin, Ras, and p53 mutation status) to SF2 in a panel of 48 human cancer cell lines. The top 500 genes were selected and interconnected in a biological network with a systems biology approach [31]. The 10 hub genes selected for the RSI algorithm were found to be the most connected genes in the network. Thus, the criteria for gene selection are based on the individual ability of the gene to predict SF2 and its biological importance within the network. The second step used the 10 genes to train a gene expression algorithm to predict SF2. This final algorithm is RSI and has been locked since the first validation studies in rectal cancer [32].

This model predicts RSI to be directly proportional to tumor radioresistance (RSI, high index = radioresistance). Prior work has shown RSI to be predictive for the benefit of radiotherapy in a number of different primary cancers, including esophageal, rectal, head and neck, breast, glioblastoma, pancreas, and prostate malignancies as well as colon and liver metastases [32–40]. RSI has correlated to outcomes of local control and overall survival in these disease sites. Since RSI is a radiation specific marker, we found RSI to be correlated to endpoints of local control and overall survival in these disease sites in patients treated with radiation but not in patients not receiving radiation therapy.

We have previously shown RSI to be prognostic in breast cancer patients treated in several independent datasets. In a dataset of patients treated at the Karolinska University Hospital (n=159), we noted patients predicted to be radiosensitive had an improved 5-year relapse-free survival when compared with radioresistant patients (95% versus 75%, p = 0.02) [33]. Since RSI is a radiation specific signature, there was no difference in radiosensitive/radioresistant patients treated without RT (71% versus 77%, p = 0.67). In addition, in a separate dataset of patients treated from the Erasmus Medical Center (n=344), radiation treated radiosensitive patients had an improved 5-year distant metastasis-free survival over radioresistant patients (77% versus 64%, P= 0.0409), but no difference was observed in patients treated without radiation (radiosensitive versus radioresistant, 80% versus 81%, p = 0.94) [33]. RSI has also been validated in a cohort of 343 patients treated at 4 Dutch Centers (Netherlands Cancer Institute, Radboud University Medical Center, Erasmus Medical Center, and Ziekenhuis Amstelland) with breast conserving therapy that included whole breast radiation with or without a tumor bed boost [37]. We noted that local recurrence was not predicted across the entire cohort. However, the combination of receptor type with radioresistance according to RSI identified a subpopulation of patients with an increased risk of local recurrence. In contrast, integrating RSI into the luminal subtypes did not identify additional risk groups with increased risk of local recurrence.

## 5. The Genomic Adjusted Radiation Dose (GARD)

**Figure 2:**
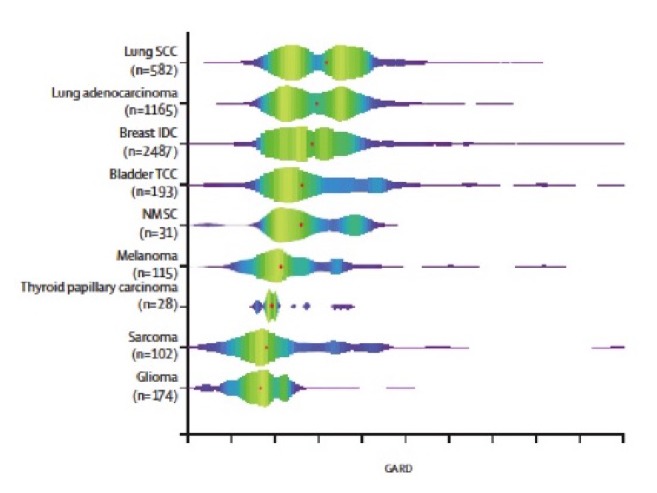
GARD score distribution and density within 60 Gy dose level, by disease site. The red dot represents the median GARD value for each disease site at assigned dose levels. Colors in the plot correlate with the sample density. GARD=genomic adjusted radiation dose. IDC=invasive ductal carcinoma. TCC=transitional cell carcinoma. NMSC=nonmelanoma skin cancer. Reprinted from* The Lancet Oncology*, Vol. 18, Scott JG, Berglund A, Schell MJ et al., A genome-based model for adjusting radiotherapy dose (GARD): a retrospective, cohort-based study, 202-211, 2017, with permission from Elsevier.

The linear quadratic model is a commonly used metric by radiation oncologists to quantify the biologic effect of radiation dose on various tumor types as well as normal tissue [41]. Since the model was developed as a molecular estimate of SF2, our group hypothesized RSI could be integrated into the model to represent a tumor's unique response to radiation therapy. Such a model, which integrates tumor specific biology into the response to radiation, is vital for progress towards precision radiation oncology. The result was the genomically adjusted radiation dose or GARD, which has since been published and validated in independent datasets of breast, glioblastoma, pancreas, and lung tumors [42]. We assessed GARD for 8,271 tumor samples in the Moffitt Total Cancer Care Database, our institution's tissue biorepository [43]. GARD values varied based on primary tumor histology (Figure 2). Median GARD values were lowest for tumor types traditionally thought to be more radioresistant including glioblastoma and sarcoma and higher for tumor types thought to be more radiosensitive including virally associated cervical cancer as well as oropharyngeal cancer.

Similarly, we have shown RSI to be correlated with the 12-Chemokine (12-CK) signature, a validated gene expression signature for immune-related and inflammation related genes[44–46]. We found the two signatures to be inversely correlated across tumor types indicating greater immune systemic activation to be correlated with radiosensitivity [47]. The 12-CK score has also been assessed in breast cancer samples at our institution [48]. Higher 12-CK scores (immune active) were noted in white patients, poorly differentiated, and basal and Her 2+ molecular subtypes. Higher 12-CK scores also demonstrated superior overall survival (p=0.008) and recurrence-free survival (p<0.0001) especially in basal and Her 2+ patients.

GARD predicted distant metastasis-free survival and relapse-free survival on multivariate analysis in two independent datasets of patients treated with adjuvant breast radiotherapy. In addition, in a separate analysis from our group presented in abstract form and currently in preparation for publication, GARD predicted for local recurrence in patients with ER negative tumors but not ER positive tumors [49]. In this analysis, we note a small subset of women who may benefit from dose escalation to the whole breast to 60 Gy reaching an optimal GARD level to compensate for an unfavorable genetic profile. These data suggest uniform dosing may lead to inferior outcomes in select populations of patients. A dosing optimization strategy, which takes into account a tumor's unique genetic profile, may allow for improved outcomes with dose escalation and potential sparing of toxicity with dose deescalation in more favorable profile tumors. Although further prospective validation is required, the model provides a framework to integrate a genomic component into the assessment of radiation effect to assess a tumor's unique sensitivity to radiation.

## 6. Immune Infiltrates and Radiation Response

It is evident the response to RT varies widely across tumors, which is partially driven by topographic biophysical variability in the microenvironment as well as the intrinsic mutational profile in individual tumor cells. Breast cancers are composed of tumor cells and a diverse mixture of stromal cells, including fibroblasts, endothelial, and innate or adaptive immune cells [50]. Each of these cellular constituents contributes to the dynamic framework of the tumor, which either promotes or antagonizes further cancer progression. Recently, immune cells have received considerable attention in oncology due to the sustainable clinical responses observed after altering the host immune response to various malignancies [51]. Whether breast cancer patients will benefit from immunomodulating strategies is an area of active investigation.

Breast cancer has traditionally been considered a more immunologically silent tumor relative to other malignancies (e.g., melanoma) due to lower observed mutational burden [52, 53], origination in a tissue that requires strict immune regulation during dynamic remodeling cycles [54], and the absence of a higher incidence in immunosuppressed individuals [55]. Though these features suggest an immunosuppressive microenvironment, decades of literature have qualitatively described tumor-infiltrating lymphocytes (TILs) in breast cancer specimens [56]. Immune cells appear to increase in density along the spectrum of breast atypia to overt malignancy [57] with the latter being further influenced by the specific breast cancer subtype [58]. As our knowledge has continued to progress, it has become more evident that not only the proportion, but also the type of TIL(s) present in breast cancer is important in determining clinical outcomes. For instance, a high CD8-positive T cell infiltrate may predict for improved clinical outcome whereas high levels of T-regulatory cells may portend to worse tumor control [59]. Most studies have associated clinical outcomes with individual immune cells or small subsets, which does not fully recapitulate the diversity of immune cells orchestrating the immune response to a tumor [60]. More recently, advancements in molecular and computational biology have facilitated a higher resolution of the immune landscape in breast cancer. Ali et al. profiled 11,000 breast tumors for 22 different immune cell types by gene expression analysis and identified that there were baseline differences in TIL presence between ER+ and ER- tumors and that the prognostic value of a given TIL differed between hormone receptor statuses [61]. Similarly, analysis of 7,270 nonmetastatic breast tumor samples demonstrated different TIL types predict differences in clinical outcomes, which is strongly influenced by breast cancer subtypes [62]. In this study, increased presence of *γδ* T cells and M1-polarized macrophages resulted in improved tumor control in ER+ tumors, whereas HER2-enriched tumors with elevated T-regulatory cells had worse tumor control. These results underscore the importance of stratifying breast tumors by subtype and immune cell composition when attempting to design rational therapeutic combinations that alter the immune response to a given tumor.

The relationship between radiation therapy and the immune system continues to evolve. Currently, it is believed radiation causes an immunogenic cell death, which is characterized by the release of various proinflammatory cytokines as well as cell autonomous ‘danger-signals' that cause TIL infiltration and tumor remodeling [63]. Consequently, radiation-induced tumor antigen release can assist in generating an ‘*in situ*' tumor vaccine [64]. Furthermore, the effects of radiation therapy may extend beyond the targeted tumor by a phenomenon termed the abscopal effect [65–67]. During this process, immune cell cross-reactivity may occur between the targeted tumor and distant tumor deposits outside the radiation field, thus potentially amplifying the systemic antitumor response.

Clinical decision-making tools that incorporate the immune composition of individual breast tumors with RSI/GARD may reveal optimal therapeutic approaches for both primary and oligometastatic disease. For example, if a given tumor has a low proportion of antitumor TILs, then radiation delivery may enhance the repertoire of responding immune cells and can be delivered up to an optimal GARD. Alternatively, if the tumor has a high proportion of antitumor TILs, then systemic delivery of an immune-modulating drug may be more appropriate as delivery of radiation to an immune-primed tumor may inadvertently destroy any present immune effectors due to their intrinsic radiosensitivity [68].

## 7. Immunotherapy Trials

A number of clinical trials are currently underway to assess the utility of immune checkpoint inhibitors in the management of breast cancer. These immunomodulators may have particular promise in the management of triple negative tumors. These tumors have been shown to have higher levels of PD-L1 expression than other breast subtypes [69, 70]. KEYNOTE-012 provided initial clinical activity and feasibility data on the use of pembrolizumab in heavily pretreated recurrent or metastatic triple negative breast cancer [71]. Response rates of 18.5% in 27 evaluable patients were noted. The median time to response in this cohort was 17.5 weeks and the median duration of response has not yet been reached. Combining radiation therapy with immune checkpoint inhibitors may hold promise [72, 73]. There is strong preclinical rationale for synergy in a combined modality approach including upregulation of PD-L1 expression and enhancement of the immunogenicity of these tumor types [74]. In addition, there is clinical evidence to suggest oligometastatic breast cancer patients treated with high dose per fraction radiation have improved overall survival as well as duration of responses compared to other solid tumor histologies [75]. Fractionated and high dose per fraction radiation may also be an optimal regimen to stimulate the immune system based on preclinical evidence [76].

There are ongoing efforts to improve upon the response rate of KEYNOTE-012 by utilizing high dose per fraction radiation. A trial has demonstrated the feasibility of combined extracranial hypofractionated radiation with pembrolizumab with assessments of response rates to be reported (NCT02730130). There is the possibility that combining data gathered with RSI and tailoring radiation therapy dose could lead to an improved ability to stimulate the immune system and the immunogenicity of immune checkpoint inhibitors. Numerous efforts are ongoing in a number of malignancies to combine radiation therapy and immune checkpoint inhibitors. A variety of different dosing schedules are being used in these trials. Although data from these ongoing trials will be available in the near future, an informed genomic approach to rationally combine these modalities with optimal sequencing and radiation dosing would be highly informative for future trial design.

## 8. Conclusion

Radiation therapy has long played an integral role in the management of breast cancer. Although for many years radiation oncologists have delivered uniform doses of radiotherapy based on long-term prospective data, an improved understanding of tumor biology as well as access to genomic information may allow for greater personalization of radiotherapy dosing in the near future. Various fractionation schedules have now shown equivalence in data and we are now at a point that, through genomics, strides can be made towards the personalization of radiation treatment delivery for the management of breast cancer. This has the potential to not only decrease treatment burden and side effects of treatment but also decrease recurrence rates.
